# Soil moisture controls the partitioning of carbon stocks across a managed boreal forest landscape

**DOI:** 10.1038/s41598-023-42091-4

**Published:** 2023-09-09

**Authors:** Johannes Larson, Jörgen Wallerman, Matthias Peichl, Hjalmar Laudon

**Affiliations:** 1 Department of Forest Ecology and Management, Swedish University of Agricultural Sciences, 901 83 Umeå, Sweden; 2 Department of Forest Resource Management, Swedish University of Agricultural Sciences, 901 83 Umeå, Sweden

**Keywords:** Forest ecology, Boreal ecology, Carbon cycle, Forestry

## Abstract

Boreal forests sequester and store vast carbon (C) pools that may be subject to significant feedback effects induced by climatic warming. The boreal landscape consists of a mosaic of forests and peatlands with wide variation in total C stocks, making it important to understand the factors controlling C pool sizes in different ecosystems. We therefore quantified the total C stocks in the organic layer, mineral soil, and tree biomass in 430 plots across a 68 km^2^ boreal catchment. The organic layer held the largest C pool, accounting for 39% of the total C storage; tree and mineral C pools accounted for 38% and 23%, respectively. The size of the soil C pool was positively related to modelled soil moisture conditions, especially in the organic soil layer (R^2^ = 0.50). Conversely, the tree C pool exhibited a unimodal relationship: storage was highest under intermediate wetness conditions. The magnitude and variation in the total soil C stocks observed in this work were comparable to those found at the national level in Sweden, suggesting that C accumulation in boreal landscapes is more sensitive to local variation resulting primarily from differences in soil moisture conditions than to regional differences in climate, nitrogen deposition, and parent material.

## Introduction

Forests provide many life-sustaining ecosystem services. It has been suggested that management interventions in forest ecosystems could be among the most effective nature-based solutions combating climate change^1,2^ because forests play critical roles in global carbon (C) sequestration and long-term carbon storage^3^. Boreal forest landscapes store approximately one third of the entire terrestrial C pool^4^, with the majority of this C being stored below ground as soil organic carbon (SOC)^5^. Various biomass components including tree trunks, branches, roots, foliage, and deadwood also hold large C pools^6^. However, the relative sizes of these above- and belowground C pools within boreal landscapes are rather poorly constrained. Global, national, and regional estimates of boreal forest C stocks are often associated with large uncertainties^7^, which are typically attributed to under-sampled regions, a lack of remote sensing data, and differences in sampling methods and intensities between studies^4^. This limits our ability to develop strategies for improving the carbon sequestration potential of forest landscapes.

It is well established that soil forming factors are sensitive to climate, time, organisms, parent material and topography^8^, all of which by extension influence the development of the SOC pool. Several studies have identified climate as a key driver of SOC accumulation on global and regional scales, mainly because of its impact on temperature and precipitation^9,10^. However, on smaller landscape scales (up to several tens of km^2^), site-specific soil-forming factors such as local topography may be more important because some of the factors mentioned above can be considered constant and are thus controlled for in small scale observational studies^11^. For example, in mountainous landscapes where the parent material can be assumed to be constant, the spatial variation in the SOC stock is largely regulated by differences in altitude and aspect that have large control on climatic variability^12^.

The central role of local topography as a primary controlling factor of soil moisture conditions is particularly evident in boreal landscapes, which are often dominated by unsorted glacial till with limited variation in hydrological properties^13–15^. Soil moisture is a major factor governing SOC accumulation^16–18^ because it influences the input of organic carbon via its effects on plant production and also controls decomposition rates. The accumulation of the aboveground C stock in boreal landscapes is also sensitive to disturbances such as fires and forest management^19^, while forest productivity is tightly constrained by climate, nutrient availability, and water levels^20,21^. Specifically, tree growth in dry sites is often limited by water and nutrient availability^22^, whereas excessive wetness leads to soil saturation and limits tree growth by creating anoxic conditions that are often associated with increased organic layer thickness^23,24^.

Managed boreal landscapes are particularly heterogeneous in terms of vegetation structure and composition, which can enhance variation in C stocks across smaller spatial scales. However, the lack of spatially extensive soil moisture data means that the landscape-scale effects of management on C stocks are poorly constrained^11^. This is a significant problem because climatic change is likely to change the water balance in boreal landscapes and thereby affect soil moisture conditions. Consequently, there is a clear need to improve our understanding of the size and distribution of C stocks on the landscape scale and to identify the factors governing them in order to develop sustainable forest management strategies.

To address these needs, we conducted a comprehensive forest and soil survey across a 68 km^2^ managed boreal forest catchment in Northern Sweden with the aim of quantifying the magnitude and variation of forest ecosystem C stocks. We sampled 430 plots, obtaining detailed soil profile descriptions of organic and inorganic soils down to 50 cm in the mineral soil and performing chemical analyses of samples from fixed soil depths. The soil survey was combined with an extensive forest survey using the same survey grid and a high resolution airborne laser scanning (ALS) dataset. Recent advances in ALS have made it possible to retrieve various forest biophysical properties^25^ and acquire high resolution topographic information, opening up new approaches to soil moisture modelling and digital soil mapping. For example, in Sweden ALS-derived topographical information has been combined with additional geographical datasets to model soil moisture conditions at a spatial resolution of 2 m using machine learning algorithms^26^. This approach was shown to accurately delineate peat soils^27^. Furthermore, high resolution estimates of above- and belowground biomass have been obtained by combining ALS and forest survey data^28–30^. These developments offer new ways to identify factors controlling the magnitude and variation of above- and below-ground forest ecosystem carbon stocks.

The specific objectives of this study were to (i) estimate the size and spatial variation of C stocks in soil and trees in a managed boreal forest landscape, (ii) characterize the relationships between the sizes of these C stocks and soil moisture conditions (iii) and produce high-resolution wall-to-wall estimates of soil and tree C stocks within the landscape. We hypothesised that (i) soil C is the largest and most variable C pool across the landscape, (ii) soil moisture conditions control SOC levels at the landscape scale, with increased soil moisture being associated with larger SOC stocks, and (iii) soil moisture effects on the organic layer C pool are a key determinant of the studied landscape’s total C stock.

## Methods

### Site description

This study was conducted in the Krycklan catchment, situated in northern Sweden (Lat. 64°,23′N, Long. 19°,78′E)^31^. The catchment has a cold temperate humid climate with a 30 year (1991–2020) mean annual air temperature of 2.4 ± 0.3 °C and a mean annual precipitation of 638 ± 40 mm, of which 35% falls as snow. The catchment spans 68 km^2^ and has a gentle topography, with elevations ranging from 127 to 372 m.a.s.l. and a poorly weathered gneiss bedrock. The soils of the upper parts are dominated by unsorted glacial till while those of the lower parts consist primarily of sorted sediments of sand and silt. Approximately 25% of the catchment has been protected for research since 1922; ownership of the remaining area is divided among private owners and forest companies. The catchment’s land cover is dominated by forests, which account for 87% of its total area and consist primarily of Scots pine (*Pinus sylvestris* L.) (63%) and Norway spruce (*Picea abies* (L.) H. Karst.) (26%). Forests in the non-protected areas are managed by conventional rotation forestry and are predominantly even-aged, artificially regenerated, and thinned. The forest soils are dominated by well-developed iron podzols^32^. Mires and lakes cover 9% and 1% of the landscape, respectively, while arable land covers 2%.

### Field data

**Figure 1:**
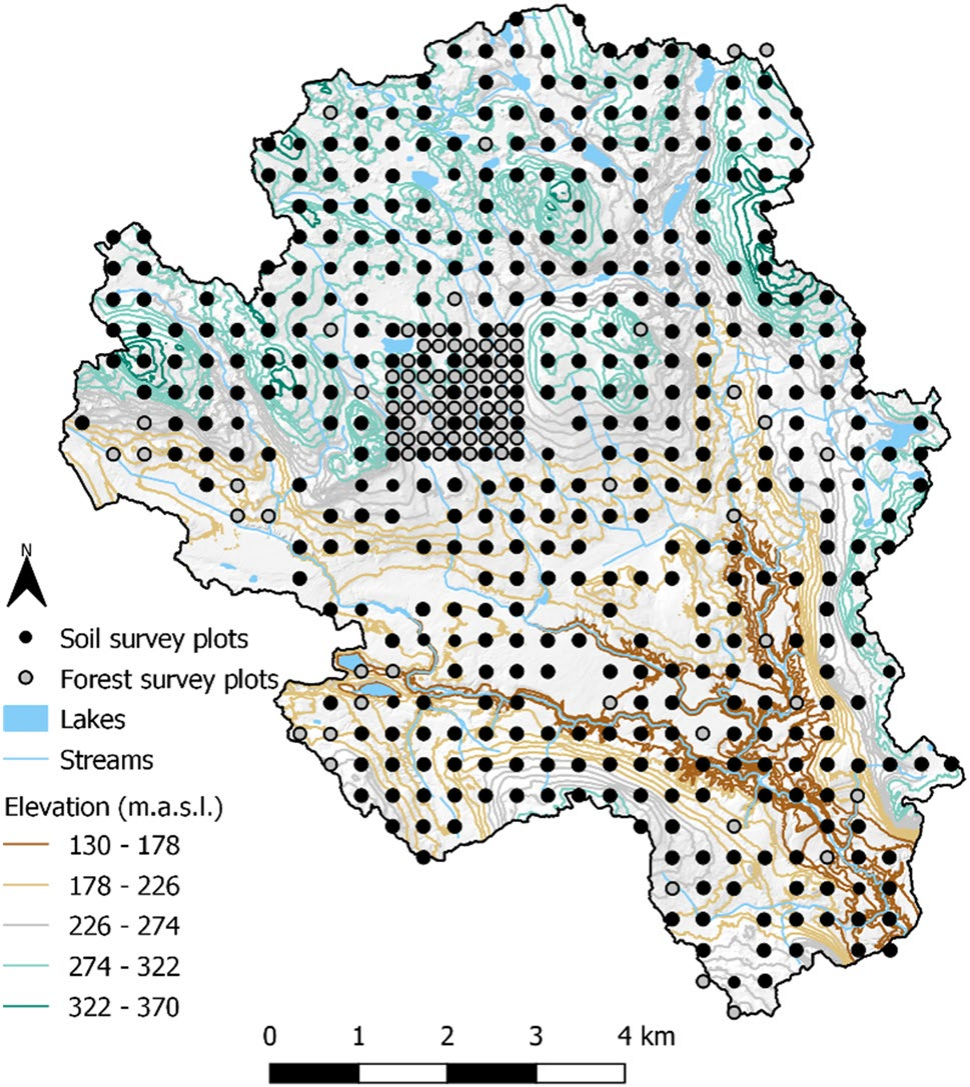
Topography of the Krycklan catchment and locations of soil and forest survey plots (represented as black and grey dots, respectively). Forest surveys were also conducted on soil survey plots. Most plots are located on the vertices of a 350 × 350 m grid but there is a densified 175 × 175 m grid around an Eddy covariance tower in the catchment’s centre. The map was created using Esri ArcGIS Pro 3.0.2, https://www.esri.com/en-us/arcgis/products/arcgis-pro/overview.

The survey grid covers the entire catchment area and consists of 500 plots that each have a radius of 10 m and an area of 314 m^2^, with a spacing of 350 m between adjacent plots (Fig. 1). The survey grid is densified in a 1500 × 1500 m area around an eddy covariance tower in the centre of the study area, where the spacing between adjacent plots is 175 m. Plot locations were established in 2015 using a randomly chosen origin and were oriented along the coordinate axis of the Swereff 99 TM projection. The centre of each plot was located in the field using a Trimble GeoXTR GNSS receiver.

### Soil survey

The soil survey was conducted during the snow-free seasons of 2019 and 2020, following the methods of the Swedish National Forest Soil Inventory (SFSI; http://www-ris.slu.se). Soil profile descriptions and site variables such as soil moisture classes (described below), humus form, organic layer thickness, and soil texture were determined, measured, or recorded for each plot. The organic layer was sampled volumetrically using a 10 cm diameter corer to the full depth of the O-horizons or to a maximum depth of 30 cm after removing the litter layer and bottom layer of mosses and carefully separating them from the mineral soil below. Samples were collected from 1 to 9 sampling points until the target sample volume of ca 1.5 L was obtained. These points were distributed within a 3.14 m^2^ subplot close to the survey plot’s centre. Mineral soil was sampled to a depth of 65 cm (or to bedrock or boulder depth) at fixed intervals of 0–10, 10–20, and 55–65 cm. Total C was analysed on the fine fraction (< 2 mm) after samples had been dried at 65 °C, ground to a fine powder and homogenised. A total of 1500 individual samples were analysed for soil C concentration by mass spectrometry using a Delta IRMS instrument coupled to a Flash EA 2000 analyzer (Thermo Fischer Scientific, Bremen, Germany). Analyses were performed with 5–50 mg soil material depending on the organic matter content. Organic layer C stocks were calculated by multiplying each sample’s C concentration by its dry weight and then dividing the result by the total sampled area. Mineral soil C stocks in each sampled layer (0–10, 10–20, and 55–65 cm) were calculated based on the C concentration, bulk density (g/cm^3^), soil layer thickness (cm) and the volume percentage of stones and boulders using the following expression:

1 $$Storage=Concentration\,\, (\%) \times BulkDens \times LayerThickness \times (100-StoneVol)/100$$

The bulk density of the mineral soil horizons was calculated using the SFSI procedure, which is based on a pedotransfer function that depends on the C concentration and depth (cm)^10,33^:

2 $$BulkDens=1.5463 \times EXP({-0.3130 \times CarbonConc}^{0.5})+0.0027 \times Depth$$

The volume of stones and boulders in each plot was estimated using the stoniness index, which is determined by driving a 1 cm diameter metal rod into the soil using a small sledge hammer (2 kg) until the rod cannot penetrate further. The penetration depth (max 30 cm) is then measured from the top of the mineral soil surface. Measurements were done at 12 predetermined locations across each plot and the volume percentage was then calculated using a transfer function^34,35^. The total SOC stock was calculated as the sum of the organic and mineral C pools. For plots with peat soils where the organic layer thickness was > 30 cm, the total C stock was calculated to a maximum depth of 1 m from the organic layer surface. In these plots, the C stock of the organic layer was estimated by collecting samples to a maximum depth of 30 cm and extrapolating downwards.

### Forest survey

The forest survey was conducted in the late fall of 2019 and the early spring of 2020. A total of 488 plots were surveyed, of which 430 were also included in the soil survey (Fig. 1). All trees within each 10 m radius plot were measured and the stem diameter at breast height (DBH; 1.3 m) of trees with DBH > 4 cm was recorded along with the heights of saplings. In regenerating/young forests and some other stands with very high stem densities, the plot radius was reduced to 5 m to limit the time needed for surveying. Species and DBH were recorded for all trees and tree heights were measured using a laser-guided hypsometer on a subjectively selected sub-sample of at least three trees that were chosen to capture the tree size variation of each species. The height of the remaining trees was estimated using plot-level fixed mixed effects modelling for single trees and then imported into the Heureka system for plot biomass calculations^36^. The aboveground biomass in each plot was estimated using allometric equations for stumps, stems, bark, dead and living branches, and foliage for Scots pine, Norway spruce, and birch, with tree height and DBH as independent variables^37^. For Lodgepole pine (*Pinus contorta* Bol.), we used the same functions as for Scots pine; other deciduous species were modelled using the birch functions. Belowground biomass was estimated for individual trees using species-specific allometric equations with DBH as the independent variable and were summarized per plot^38^. The total tree C pool was calculated by summarizing the above- and belowground biomass for each plot and then converting to Mg C ha^−1^, assuming a C concentration of 50% in biomass.

### Soil moisture classes

Each plot was assigned to one of five soil moisture classes based on its average groundwater table depth, which was estimated from the plot’s position in the landscape, soil texture, and vegetation patterns. The five soil moisture classes were: dry (7% of all plots), mesic (73%), mesic-moist (11%), moist (7%) and wet (2%). These classes are described briefly below and at greater length in previous publications^39^.

- Dry soils have an average groundwater table > 2 m below the soil surface. They tend to be coarse-textured and can be found on hills, ridges, and eskers. Dry soils are mainly Leptosols, Arenosols, Regosols, or Podzols with thin organic and bleached horizons.
- Mesic soils have an average groundwater table between 1 and 2 m below the soil surface. Podzol is the dominating soil type with a fairly thin (4–10 cm) organic mor layer covered mainly by dryland mosses (e.g., *Pleurozium schreberi*,* Hylocomium splendens* and *Dicranum scoparium*). They can be walked on dry-footed even directly after rain or shortly after snowmelt.
- Mesic-moist soils have an average groundwater table depth < 1 m below the soil surface and are normally located on flat ground in lower-lying areas or lower parts of hillslopes. The soils become wet seasonally following snowmelt or heavy rain events. The feasibility of crossing with dry feet in normal shoes depends on the season. Peat mosses (e.g., *Sphagnum *sp., *Polytrichum commune*) in patches are common, and trees often grow on humps. Podzols are commonly found but often with a thicker organic layer than in mesic sites. The organic layer is often classified as peaty mor.
- Moist soils have an average groundwater table depth < 1 m below the soil surface and the surface water is commonly visible in depressions within the plot. Moist soils are found at lower altitudes, on the lowest parts of slopes and flat areas below larger ranges. They can be crossed in shoes without getting wet feet by utilizing tussocks and higher-lying areas. The vegetation includes wetland mosses (e.g., *Sphagnum* sp., *Polytrichum commune*, *Polytrichastrum formosum*). When stepping in depressions, water should form around the feet even after dry spells. Trees often grow on small mounds and the soil type is most often Histosol, Regosol, or Gleysol.
- Wet soils have a ground water table close to the soil surface and permanent pools of surface water are common. Soils are typically Histosols or Gleysols. Drainage conditions are very bad and they cannot be crossed in shoes without getting wet feet. Wet areas are often located on open peatlands and coniferous trees seldom develop into stands.

### Modelled soil moisture conditions

Soil moisture conditions were modelled using the newly developed SLU machine learning soil moisture map with a resolution of 2 m^26^. The map was developed using multiple nationwide geographical information datasets including various terrain indices, climate data, and quaternary deposit information. The training and validation data consisted of almost 20,000 field soil moisture classifications (1–5) from the national forest inventory that were spread across the entire Swedish forested landscape. The final model used Extreme Gradient Boosting (XGBoost) to produce a 2-class model in which the depth water index^40^ and topographic wetness index^41^ were the most important predictors. The survey grid employed in the present study was used for external validation of the modelled soil moisture, which yielded a kappa value of 0.52^26^. The model’s output is presented as a wetness index map showing the predicted probability (0–100%) of wetness for each pixel and is publicly available (Swedish University of Agricultural Sciences, 2022). Modelled soil moisture conditions for each survey plot were extracted using the coordinates of the plot’s centre.

### Carbon pool mapping

Data representing all plots included in the forest survey of 2019 were used as ground truth for Tree C pool mapping. ALS data were acquired in August 2019 using a Reigl VQ-1560i-DW 1064 nm (NIR) scanning system with an average point density of 20 points m^−2^. The raw ALS data were pre-processed by classifying point returns as ground, unclassified, or noise. A digital terrain model was then generated and the ALS points were normalised to represent the tree canopy height above the ground surface. Finally, metrics were generated from the ALS data to summarize the point-cloud information on the raster cell level using the CloudMetrics program in the Fusion software package^42^. These metrics were calculated for 12.5 × 12.5 m grid cells using methods previously developed to generate ALS estimates on a national scale^43^. Plots were excluded if the absolute difference between Lorey’s mean height and the ALS metric P95 (the 95th percentile of the ALS point cloud’s height distribution) was above 5 m. Regression models relating the observed Tree C pool at the plot level to several other explanatory ALS metrics were fitted and extrapolated over the entire study area. The total SOC stocks over the catchment area were mapped using the modelled relationship between plot-level measurements of total SOC stocks and the SLU soil moisture map.

### Statistics

Descriptive statistics for the different C pools were generated using the statistical software R^44^. The relationships between modelled soil moisture conditions and C pools were evaluated by linear regression, using polynomial models in some cases. Predictive models with log-transformed dependent variables were back-transformed using smearing estimates^45^ to avoid bias. As no independent data were available to assess the accuracy of the models’ C pool predictions, we performed leave-one-out cross-validation^46^ by removing one sample from the model dataset and fitting the selected models on the remaining plots. Model performance was evaluated using R^2^ and RMSE.

## Results

### Soil carbon pools

The mean total SOC stock down to 50 cm of mineral soil including peat soils was 94 ± 5 (SE) Mg C ha^−1^ (Table 1). Excluding peat soils, the mean total C stock was 67 ± 2 Mg C ha^−1^. The mean SOC stock in mineral soils was 40 ± 1 Mg C ha^−1^ while that in the organic layer (to a maximum depth of 1 m) was 59 ± 6 Mg C ha^−1^. Forty-nine plots were classified as peat soils (organic layer thickness > 30 cm); the mean C stock for these plots was 307 ± 29 Mg C ha^−1^.

**Table 1:** Soil carbon stocks (Mg C ha^−1^).

Variable	Case	N	Mean	SD	Median	Min	Max	SE
Total SOC pool	Including peat soils	430	94	109	62	9	959	5
Organic C pool	Including peat soils	430	59	115	21	0	959	6
Mineral C pool	Including peat soils	430	35	23	35	0	171	1
Total SOC pool	Excluding peat soils	381	67	43	58	9	412	2
Organic C pool	Excluding peat soils	381	27	33	19	0	336	2
Mineral C pool	Excluding peat soils	381	40	22	37	0	171	1
Total SOC pool	Only Peat soils	49	307	198	291	21	959	29

### Tree carbon pool

The forest age varied between 0 and 272 years with a mean of 79. The mean height and basal area were 13 m and 21 m^2^ ha^−1^, respectively (Table 2). The total tree C pool varied from 0 to 228 Mg C ha^−1^, with a mean of 58 Mg C ha^−1^. On average, 24% of the Tree C was stored below ground and 76% above ground (Table 3).

**Table 2:** Field measurements of forest stand variables in the forest survey plots (n = 488).

Variable	Mean	SD	Median	Min	Max	SE
Age (years)	79	48	73	0	272	2
Hgv (m)	13	5	14	0	24	0.24
Basal area (m^2^ ha^−1^)	21	12	21	0	58	0.5
Volume (m^3^ ha^−1^)	156	111	149	0	601	5.0
Number of stems (ha^−1^)	1459	1835	1178	0	33,205	83

**Table 3:** Tree C pool stocks (Mg C ha^−1^) in the surveyed plots (n = 488).

Variable	Mean	SD	Median	Min	Max	SE
Tree C pool	58	40	55	0	228	2
Above ground	44	30	41	0	170	1
Below ground	14	10	14	0	58	1

### Total carbon stock estimates

The total SOC pool accounted for 62% (94 ± 1 Mg C ha^−1^) of the landscape’s total C storage (152 Mg C ha^−1^), with the remaining 38% (58 ± 2 Mg C ha^−1^) being stored in the tree C pool. The largest individual C pool was the organic layer (59 ± 6 Mg C ha^−1^), which comprised 39% the total C stock on average, while the mineral soil C pool accounted for 23% of the total (35 ± 1 Mg C ha^−1^). If peat soils were included, the organic soil C pool accounted for 63% of the total SOC pool. However, if peat soils were excluded, the mineral soil C pool comprised 60% of the overall SOC stock.

### Soil moisture effects on C allocation

**Figure 2:**
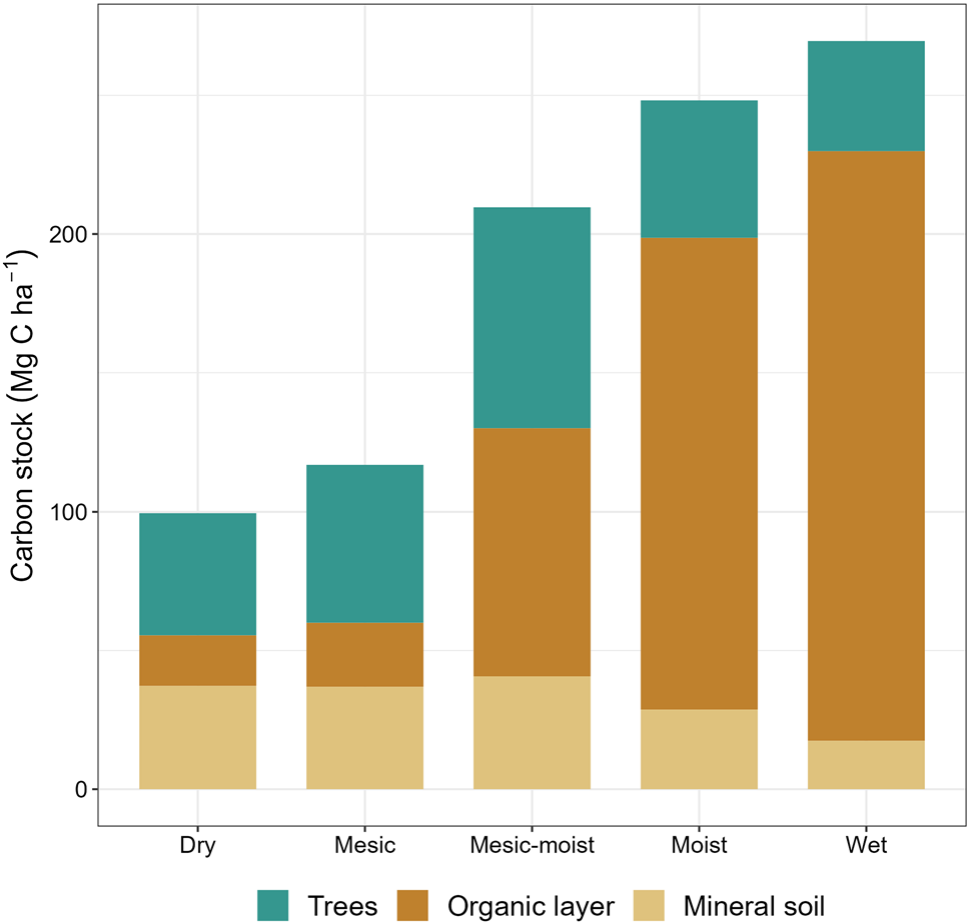
Sizes of the tree, organic layer, and mineral soil carbon pools for different field-classified soil moisture conditions.

The size of the total C pool differed significantly between soil moisture classes, ranging from 100 Mg C ha^−1^ in the driest class to 270 Mg C ha^−1^ in the wettest (Fig. 2). This relationship was mainly driven by an increase in the size of the organic layer C pool in the mesic-moist to wet soil moisture classes. The C stored in the mineral soil C pool decreased from 37 to 18 Mg C ha^−1^ between the driest and the wettest class; this is mainly due to the greater depth of the organic layer in wetter soils and the fact that sampling was only conducted to a maximum depth of 1 m below the soil surface. The mineral soil C pool depth was therefore reduced or zero in cases where the organic layer thickness was around or above 1 m. The tree C pool increased from 44 Mg C ha^−1^ in the dry class to a maximum of 80 Mg C ha^−1^ in the mesic-moist sites but then decreased as the moisture increased further, falling to 40 Mg C ha^−1^ in the wettest soil class (Fig. 2).

**Figure 3:**
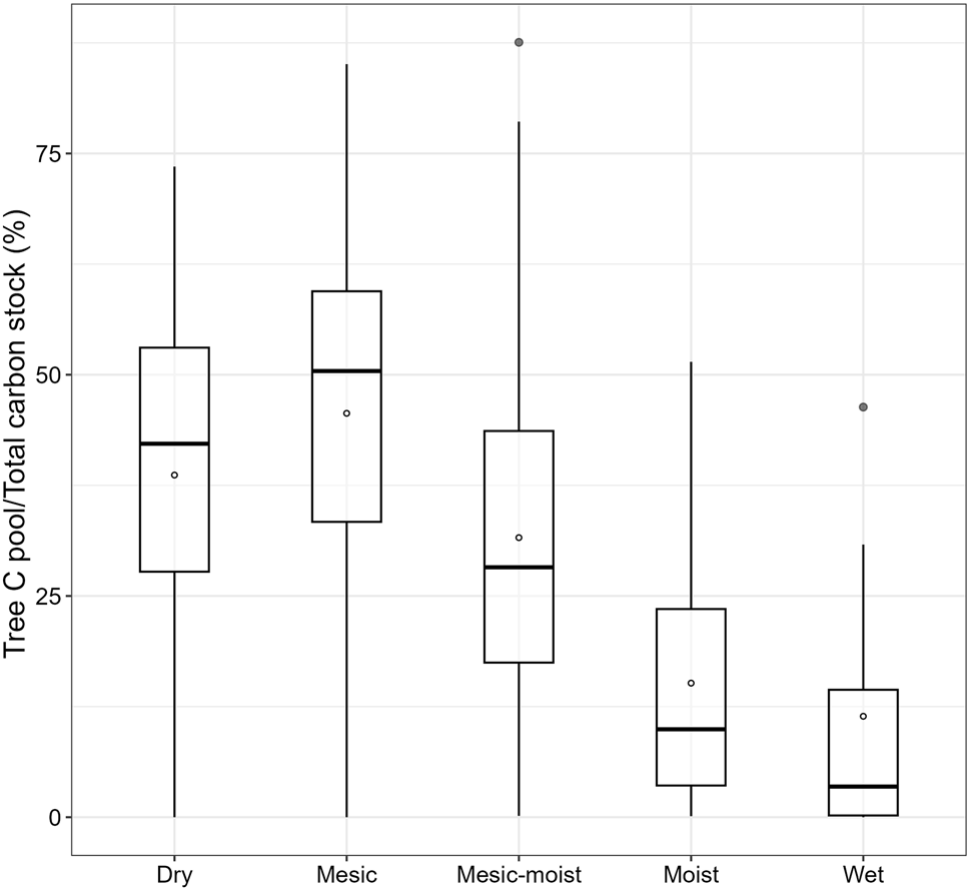
The tree carbon pool as a proportion of the total carbon stock in each of the five soil moisture classes. Mean values are indicated by white circles.

The median proportion of the total C stock in the tree C pool increased from the dry (42%) to mesic (51%) soil moisture classes (Fig. 3). The majority (57%) of the survey plots had over 50% of their total stored C in the soil.

### A model to predict C pool sizes based on soil moisture

**Figure 4:**
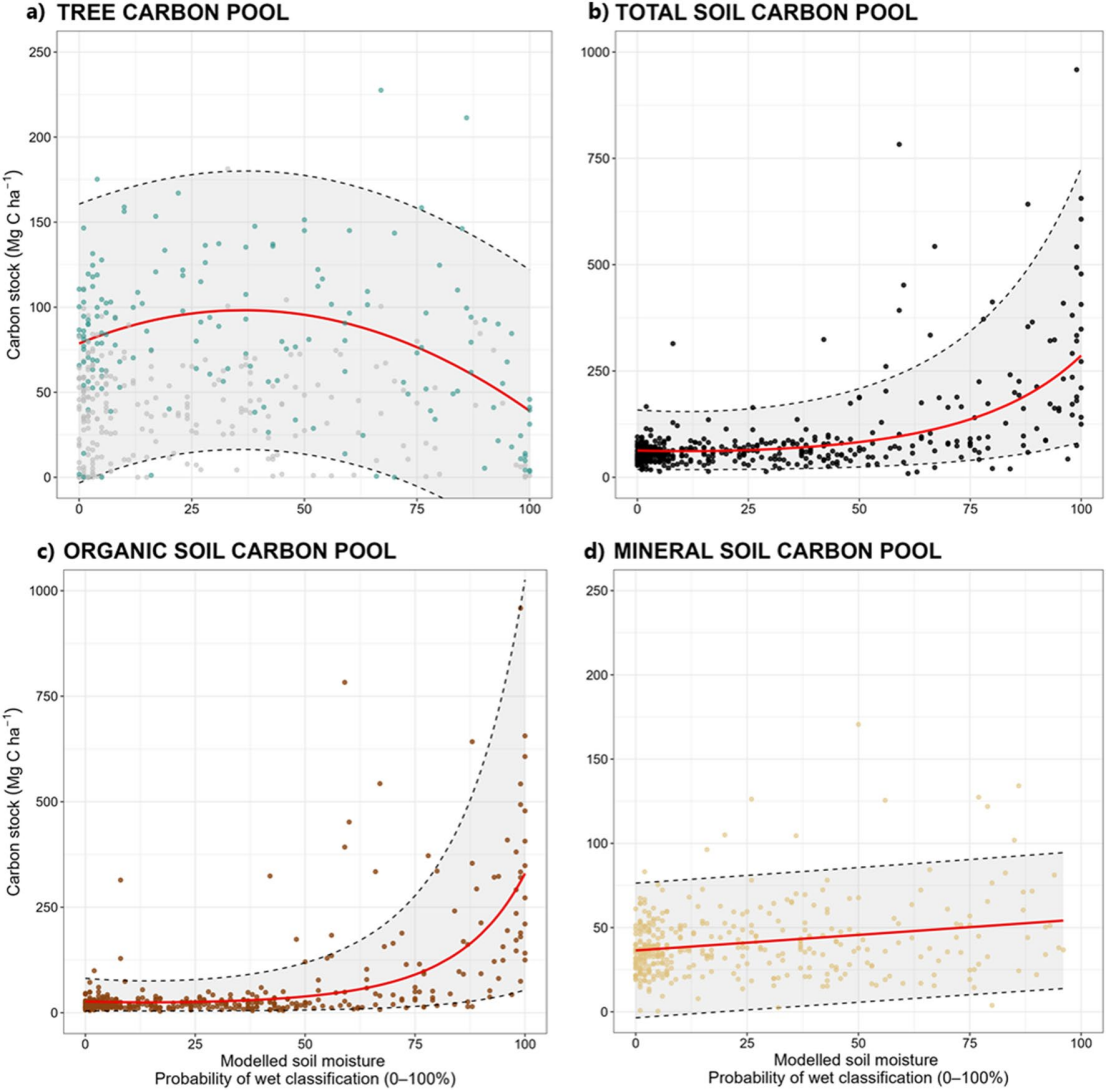
Carbon pool sizes as functions of modelled soil moisture conditions. Regression lines are shown in red and 95% prediction intervals are shown using dashed lines. The modelled soil moisture represents the probability of plots being classified as wet (dry − wet) based on a 2-class XGBoost model. The tree carbon pool modelling results shown in plot (**a**) are based on data for survey plots with a stand age of 80 years or above (results indicated by blue dots) to reduce the impact of management effects. Results for plots with a mean stand age below 80 years are represented by grey dots.

Upon relating the measured C pools to soil moisture predictions obtained using the SLU soil moisture map, we found that the relationship between the tree C and SOC pools at different soil moisture levels was unimodal (Table 4; Fig. 4), in accordance with the results obtained using the field soil moisture classifications (see Fig. 3). The relationship between the total SOC pool size and the modelled soil moisture was described well by a polynomial regression (R^2^ = 0.40)(Table 4), which accurately captured the large increase in C stocks with increasing soil moisture (Fig. 4b). This analysis also confirmed that the increase in the total SOC stock was mainly due to an increase in the size of the organic layer C pool (R^2^ = 0.50). The mineral C pool showed a significant positive linear increase with the soil moisture, but this trend explained only 5% of the total variation in C pool size.

**Table 4:** Results obtained using linear and polynomial regression models of the relationship between carbon pool size and predicted soil moisture (x). RMSE values were calculated by leave-one-out cross validation (LOOCV) in which the Total SOC and Organic SOC stocks were retransformed using SMEAR to avoid logarithmic bias.

Carbon pool	n	Regression	R^2^	RMSE	F-stat	p-value
Tree C pool_(stand age>=80)_	166	y = 80.57 − 129.3x − 165.9x^2^	0.14	40.74	13.09	5.362e−06
Total SOC pool	430	log(y) = 4.23 + 8.51x + 3.35x^2^	0.40	90.24	140.3	< 2.2e−16
Organic C pool	430	log(y) = 3.32 + 13.87x + 6.12x^2^	0.50	95.73	209.8	< 2.2e−16
Mineral C pool	373	y = 36.43 + 0.19x	0.05	20.36	24.82	7.108e−06

To avoid confounding effects from forest management on the standing biomass across our 430 plots, we also evaluated the relationship between the tree C pool and soil moisture in plots containing only tree stands that were at least 80 years old, representing mature forests (n = 166). In this analysis, the tree C pool showed a weak but significant (p-value < 0.01) unimodal relationship with the modelled soil moisture, indicating that the proportion of the total C stock in trees is generally higher in areas with intermediate soil moisture than in those with very low or very high wetness.

### Carbon mapping (wall-to-wall estimates) across the forest landscape

To map the tree C pool (including both the above- and belowground pools) over the entire catchment area, we developed a model based on the relationship between the field tree C data and ALS-derived metrics by adapting the previously-reported area-based method^47^. The final model (Eq. 3) included two dependent ALS variables relating to height distribution (P95 and SD, i.e., the 95th percentile and the standard deviation of ALS point heights above ground, respectively), and one relating to tree canopy density (VR, i.e., the proportion of ALS points reflected in the vegetation).

3 $$Tree\,\, C\,\, pool= 4.94+0.02{(P95 \times VR)}^{1.2}-3.17HSD$$

**Figure 5:**
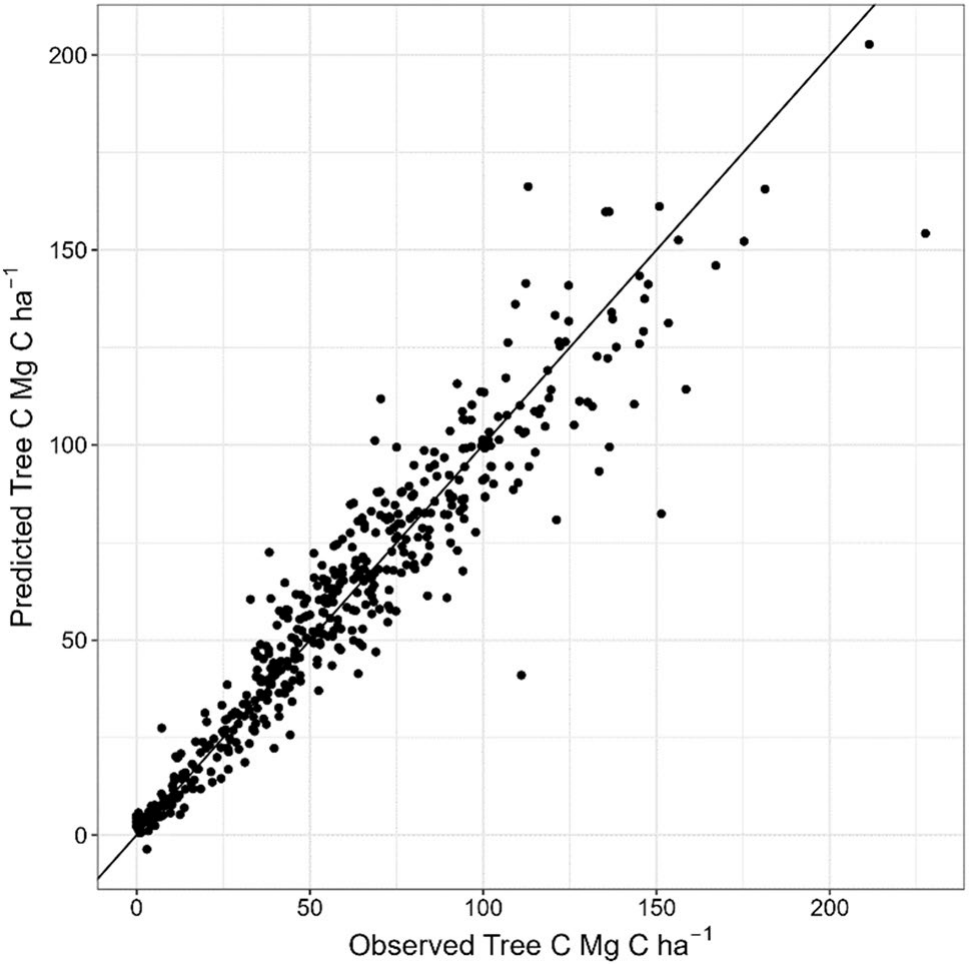
Relationship between ground truth data and the Tree C pool predicted by the ALS model (Eq. 1).

**Figure 6:**
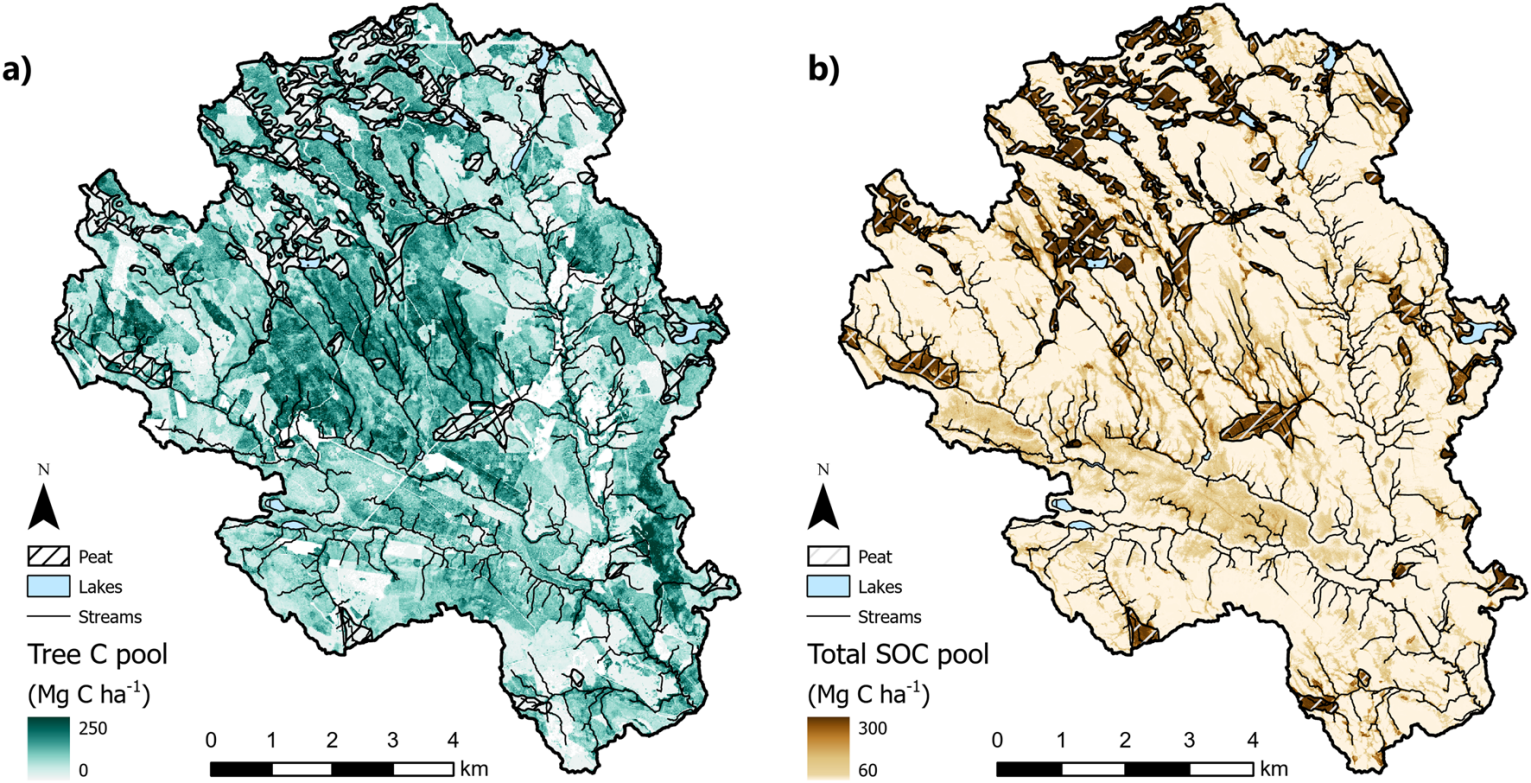
Tree C distribution map derived from ALS data using the area-based method (**a**) and the total SOC stock distribution derived by regression analysis of soil moisture data (**b**). Areas shown in white are dominated by clear-cuts and open peatland. The maps were created using Esri ArcGIS Pro 3.0.2, https://www.esri.com/en-us/arcgis/products/arcgis-pro/overview.

The agreement between the predicted and observed data was good (R^2^ = 0.9, p < 0.001) (Fig. 5), and leave-one-out cross validation indicated an acceptable goodness of fit with a RMSE of 12.4 Mg C ha^−1^. The model was therefore used to predict the tree C pool for each 12.5 × 12.5 m raster cell within the Krycklan catchment (Fig. 6a).

To map the SOC stock across the entire catchment, we applied the polynomial function described in section "A model to predict C pool sizes based on soil moisture" to each 2 m cell based on the modelled soil moisture (Table 4). This revealed a mosaic of clear cuts (white) and mature stands with high tree C stocks, demonstrating the profound effects of forest management on tree C pools within the landscape (Fig. 6a). Total SOC stocks were highest in wetlands (peat) and the riparian zones alongside streams (Fig. 6b). The inverse relationship between high soil C stocks and the size of the tree C pool was particularly pronounced in the wetland areas.

## Discussion

Despite the importance of boreal forests for carbon sequestration and climate mitigation, the factors governing C stock variation and its distribution at the landscape scales remain poorly understood. Based on a extensive survey of the tree and SOC pools in > 400 sample plots within a landscape-scale study area, this work provides (i) insights into the magnitude and variation in C stocks across a meso-scale boreal landscape; (ii) empirical evidence of the profound impact of soil moisture conditions on SOC stocks; and (iii) high-resolution estimates of the C stock distribution over a managed boreal forest landscape. Taken together, our results show how the total and individual organic and mineral SOC stocks vary across the boreal landscape and co-vary with the tree C pool.

Although we found that the total C stocks at the plot level are highly variable across a 68 km^2^ managed boreal forest landscape catchment, our estimate of the average landscape SOC stock (94 ± 3 Mg C ha^−1^) is similar to previous regional and national SOC stock estimates based on the Swedish national forest soil inventory. For instance, a national study focusing on Swedish podzols (i.e., excluding peat soil) estimated an average total SOC stock^16^ of 82 ± 3 Mg C ha^−1^. In the same study, Olsson et al. found that the average SOC pool size in the organic layer was 28 Mg C ha^−1^, which is identical to the value obtained in our analysis when peat soils were excluded. In a regional analysis covering all of northern Sweden, Hounkpatin et al. estimated a mean total SOC stock of 73 Mg C ha^−1^, which also is consistent with our results. The fact that the average SOC stocks in our boreal catchment are similar to previously reported regional- and national-scale estimates for Sweden suggests that SOC stocks are far more sensitive to local-scale variation than to differences along the national north–south gradient despite the associated wide variation in climate, nitrogen deposition, and parent material.

In accordance with our first hypothesis, the total C stock increased rapidly with the soil moisture level, primarily because of a large increase in the size of the organic layer C pool (Fig. 2). Findings from other boreal landscapes support our results: multiple studies have concluded that SOC stocks increase with soil moisture levels, whether evaluated on the basis of drainage class or wetness indices^18,48^. However, this study goes beyond previous works because it is based on a unique high-density soil dataset for a catchment-scale site; the catchment scale has received little attention in previous research. Furthermore, while organic soils often are excluded or considered separately from mineral soils due to differences in soil formation conditions, our work highlights the need to include organic soils to fully understand overall variation in C stocks in high altitude landscapes. Peat soils host a large proportion of the total terrestrial C stock in boreal biomes; our estimates suggest that they account for about one-third of the global SOC stock to a depth of 1 m^49^. Even though only 11% of the plots within this study area were peat soils, they accounted for 37% of the total measured soil C stock.

Forest management practices, particularly clear-cut harvesting, have significantly affected the natural variation of tree C stocks within boreal forest landscapes, reducing the impact of natural disturbances that previously had central roles such as forest fires and wind. The long history of forest management in Sweden has probably obscured the relationship between the tree C pool and soil moisture conditions in a way that may depend on site-specific conditions (Fig. 4). Additionally, the legacy of peatland drainage efforts within the catchment and across Fennoscandia has enhanced forest production in many areas, greatly expanding the tree C pool^50^. Evaluating these impacts can be challenging, but the successful application of our area-based method in this work clearly shows that ALS provides an effective way to systematically collect forest information in order to quantify aboveground carbon stocks on the landscape scale^29^ while also dealing with confounding factors resulting from forest management interventions (Fig. 6a).

The SOC pool accounted for a large proportion of the total C stock within our studied boreal landscape, highlighting the presence and impact of local C stock hotspots in wet peat soils (Fig. 6b). It is notable that peat soils are not only found in forested and open wetlands but also in the riparian zones lining most streams. The proportion of C stored in trees in these wet areas is substantially lower than in other forested regions, so less common management practices such as continuous-cover forestry may be preferable to ensure the preservation of these large SOC stocks^51^. More generally, the presence of large SOC stocks in riparian zones suggests a need for greater caution in forest management when dealing with such near-stream areas^52^.

To better understand the landscape-scale variation in SOC stocks, the effects of factors such as forest productivity, management, tree species, and fire history will have to be studied. Future work should also focus on exploring the combined impacts of different soil forming factors across fine spatial scales, including soil texture, bulk density, soil depth, and chemical properties. Special attention should be given to improving the reliability of bulk density estimates for unsorted sediment soils because quantifying uncertainty in this area is difficult and time-consuming. Following the method of the Swedish national forest inventory, we modelled bulk density in the mineral soil using empirical pedotransfer functions; this represents a notable weakness in our C stock estimates given the limited accuracy of such functions. Furthermore, we chose to focus specifically on testing soil moisture effects by using a state-of-the-art map based on terrain indices and other geographical information in this study^26^. However to better understand the influence of topography as a soil forming factor we could also consider the C stock in relation to individual terrain indices such as the commonly used Topographic Wetness Index (TWI)^41^ and the associated effects on aboveground productivity and soil chemical properties.

## Conclusion

We have presented a unique perspective on the total C stock of a managed boreal forest landscape that emphasizes the importance of soil moisture conditions as a key regulator of the SOC stock distribution. Our results indicate that the total C stock increases when moving from dry to wet areas, but the tree C stock is highest in regions with intermediate soil moisture levels. Landscape-scale soil moisture variation is largely governed by topography because it controls the distribution of water, which determines the spatial distribution of different soil types. To clarify the distribution and dynamics of the above- and belowground C pools, future studies should focus on disentangling the multiple drivers of C accumulation such as ecosystem productivity, species, forest history and other soil forming factors. Our results also indicate that potentially drier future conditions due to climate change might reduce the total landscape C storage and shift its allocation from soils towards tree biomass. This would have important implications for the C pool’s protection from disturbances (e.g., fire and wind throw) and associated risk of terrestrial C being emitted to the atmosphere.

## Data Availability

The dataset generated during the current study is available from the corresponding author on reasonable request.
